# Research highlights for issue 3

**DOI:** 10.1111/eva.12153

**Published:** 2014-03-17

**Authors:** Britt Koskella

**Affiliations:** Research highlights associate editor Evolutionary Applications

Whether evolution is predictable becomes a key question when deciding how to translate and apply evolutionary theory to solve real-world problems. Mutations arise by chance, and therefore, it seems fair to question if we can predict when, where, and how a population will respond to a given selection pressure. One of the many exciting results from Richard Lenski's long-term experimental evolution of *Escherichia coli* bacterial populations was the clear demonstration that, given a set of so-called ‘potentiating’ mutations, populations followed the same evolutionary trajectory again and again as the experiment was ‘replayed^’^ (Blount et al. 2008). In their new paper, Diana Blank and collaborators have taken a similar approach by experimentally evolving *E. coli* mutants with major gene deletions to test if and how the mutants are able to regain function (Blank et al. 2014). By measuring both the likelihood of recovery and the type of mutations underlying recovered function, the authors uncover a relationship between regulatory versus structural mutations and metabolic function, demonstrating a clear and predictable link between molecular change and functional innovation.

The ability to predict how a population will respond to selection pressure is further aided by a good understanding of the ‘fitness landscape’ (the relationship between genotype and reproductive success). If the effect of every possible mutation, and interactions among mutations, on an organism's fitness was known, we would have a much higher probability of successfully predicting the evolutionary trajectory in response to a given selection pressure such as drug treatment or insecticide use. Recent work by Ashley Acevedo and colleagues used next-generation sequencing to reveal the mutation landscape of poliovirus and to explore the fitness change associated with thousands of mutations across the landscape (Acevedo et al. 2014). This work sets the stage for generating and testing predictions regarding the evolution of RNA viruses, taking us a step closer to designing ‘evolution-proof’ treatment strategies.

The predictability of evolution at the phenotypic level can also be observed in natural populations, in particular when multiple populations are evolving in response to similar selection pressure. For example, a recent study by Rüdiger Riesch and coauthors examined change across nine species of New World livebearing fish that independently colonized toxic, sulfur spring environments (Riesch et al. 2014). They discovered that, as predicted by evolutionary theory, each of the 22 populations evolved toward larger offspring size and fewer offspring number after colonization, emphasizing that parallel adaptation to a specific environment can lead to repeatable evolution at the phenotypic level across both populations and species.

Finally, a good working knowledge of the mutational landscape and genotype–phenotype map can be extremely useful when employing adaptive laboratory evolution to generate organisms of applied interest. Artificial selection has been around since prehistoric times, but recent advances in, for example, stress-induced and transposon mutagenesis as well as experimental evolution have allowed for the rapid generation of organisms with desired traits. These techniques have recently been employed to select for acid-and temperature-tolerant strains of hydrogen-producing photosynthetic bacteria (Cai and Wang 2014) improved carotenoid production in yeast (Reyes et al. 2014) and reduced ethanol content of fermenting wine (Tilloy et al. 2014). For example, 200 generations of artificial selection for reduced ethanol and enhanced glycerol yields of *Saccharomyces cerevisiae* were enough to generate new strains showing little to no decrease in fermentation activity (Tilloy et al. 2014). In comparison with the speed at which mutants can be generated by engineering and mutagenesis (e.g., Cai and Wang 2014), this method may seem old-fashioned. However, Valentin Tilloy and collaborators argue that given the feasibility of classic methods and the public hesitation surrounding genetic modification, artificial selection still holds great promise for the generation of commercially relevant organisms (Tilloy et al. 2014). The success of such ‘directed evolution’ will of course depend on our ability to choose the appropriate selective pressures, as emphasized by Luis Reyes and coauthors who recently evolved yeast strains under oxidative stress with the goal of selecting for increased production of carotenoids with known antioxidant properties (Reyes et al. 2014).

These recent studies highlight that our ever-increasing understanding of evolutionary trajectories along the fitness landscape, uncovered by the genotype–phenotype map, is allowing both for an increased ability to predict the outcome of evolution and for the improved application of directed evolution in generating desirable phenotypes. Furthermore, the use of next-generation sequencing to generate single nucleotide fitness landscapes will offer a resolution that was once only dreamed of (Wright 1932).
